# Supplementary figures and images for: Organic eutrophication increases resistance of the pulsating soft coral Xenia umbellata to warming
Source: PeerJ. 2020 Jun 22;8:e9182. doi: 10.7717/peerj.9182 (PMC7316076; doi:10.7717/peerj.9182)

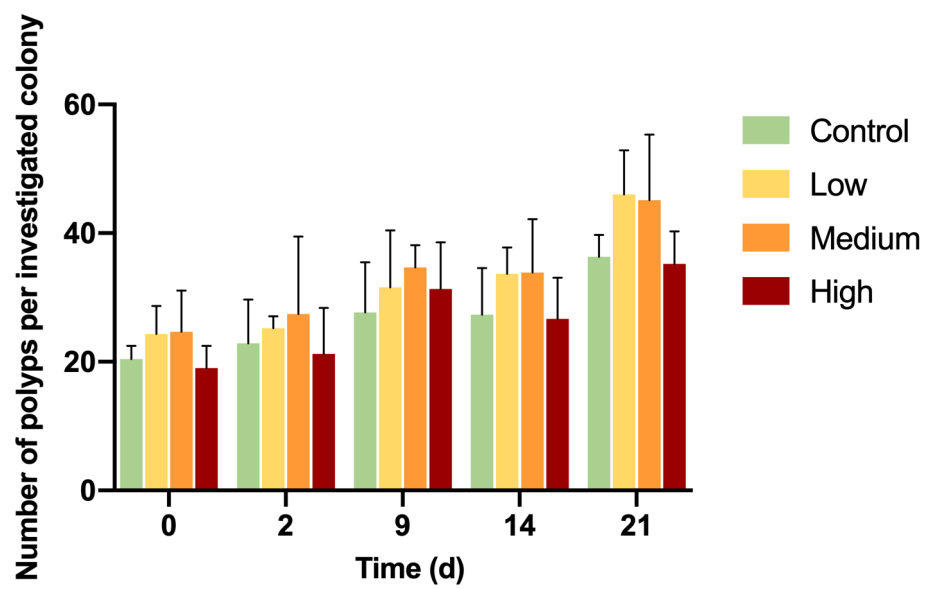

Supplement: Supplemental Information 1 — Columns indicate mean values of three replicates with error bars providing the respective SD. [file peerj-08-9182-s001.pdf]

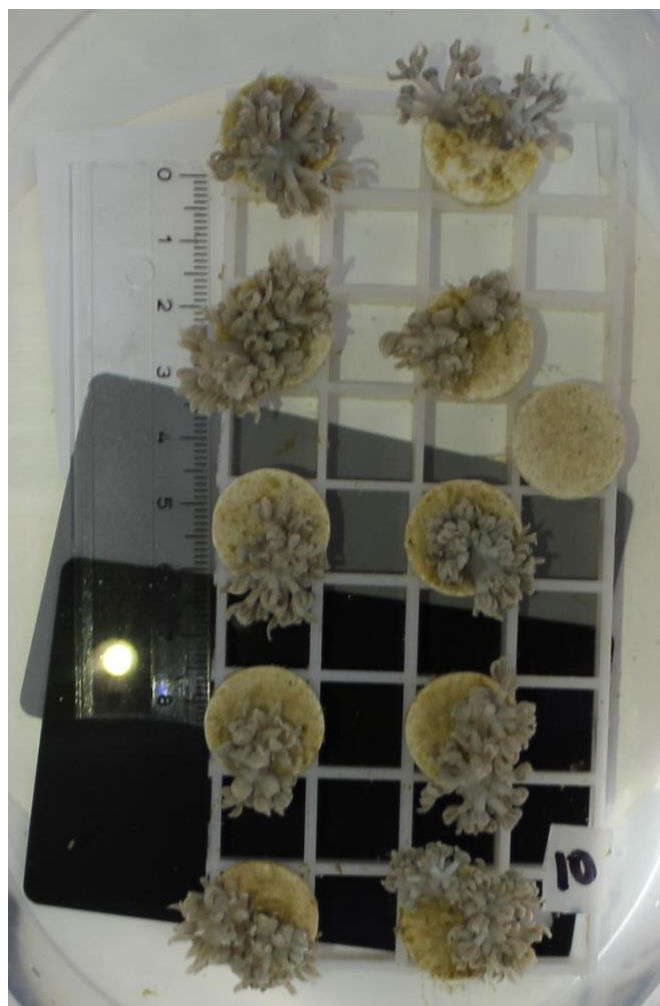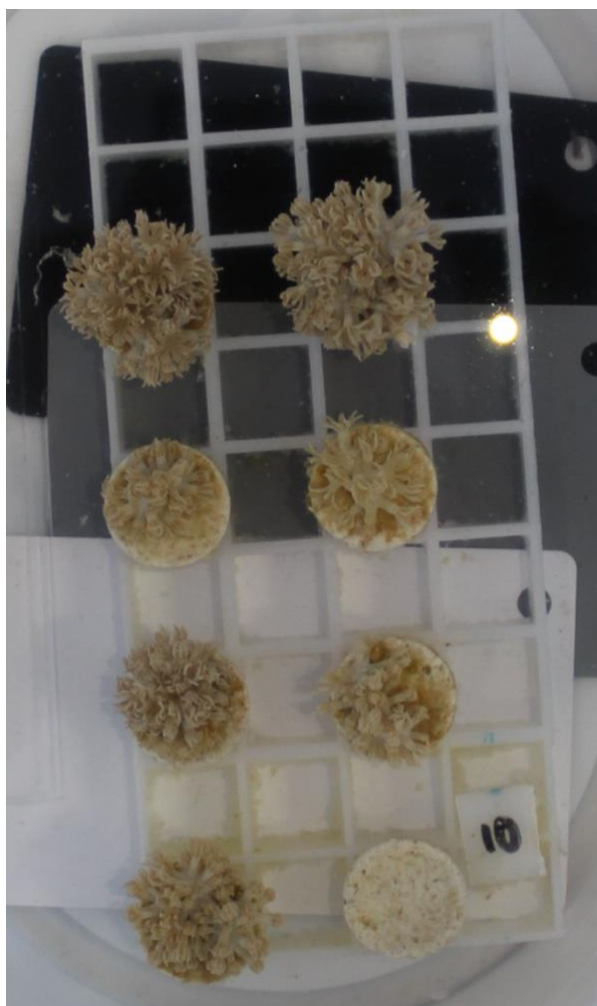

Supplement: Supplemental Information 2 [file peerj-08-9182-s002.pdf]
